# Case report: Long-term survival in synchronous double primary malignancies of lung adenocarcinomas and esophageal squamous cell carcinoma treated with definitive chemoradiotherapy and SBRT combined with anti-PD-1

**DOI:** 10.3389/fimmu.2025.1548176

**Published:** 2025-02-14

**Authors:** Rui Zeng, Xiaoyun Zhou, Kexin Ou, Wei Chen, Chen Yang, Ting Wang, Yani Li, Yawen Zha, Minying Li, Jingjing Zhang

**Affiliations:** ^1^ The First Clinical Medical College, Guangdong Medical University, Zhanjiang, Guangdong, China; ^2^ Department of Radiotherapy, People’s Hospital of Zhongshan, Zhongshan, Guangdong, China; ^3^ Department of Radiation Oncology, Shenzhen University Medical School, Shenzhen, Guangdong, China

**Keywords:** double primary malignancies, esophageal squamous cell carcinoma, lung adenocarcinomas, radiotherapy, anti-PD-1

## Abstract

**Background:**

The occurrence of multiple primary cancers has become common, and the treatment of such patients is very complex, so it is necessary to combine a variety of individualized treatment methods to achieve better treatment results.

**Case description:**

This report describes a patient with double primary tumors of lung and esophageal cancer had more than 36 months survival with non-operation treatment. The patient diagnosed as lung adenocarcinomas (LADC) and esophageal squamous cell carcinoma (ESCC), was treated with albumin-bound paclitaxel, nedaplatin, and anti-programmed death 1 (anti-PD-1). The esophageal lesions achieved complete response (CR) after finishing two courses of induction chemotherapy combined with anti-PD-1 followed by definitive chemoradiotherapy (CRT). Radiation pneumonitis (RP) occurred one month after the completion of CRT. The pneumonia was relieved after dexamethasone and moxifloxacin treatment. Then, the lung lesion was treated with oral chemotherapy followed by stereotactic body radiation therapy (SBRT). As of July 2024, the patient has survived for more than 3 years after the above treatments, and the current efficacy evaluation is CR of esophageal lesions, PR of pulmonary lesions.

**Conclusion:**

The multi-modality approach of systemic therapy combined with localized radiotherapy is an effective treatment in the patients of the double primary malignant tumors of LADC and ESCC. The safety and toxicity of radiotherapy for the thoracic double primary tumors demonstrate acceptability.

## 1 Introduction

Lung and esophageal cancers are among the most common malignant tumors worldwide (1). However, cases of synchronous double primary lung and esophageal cancers are rarely reported, and the absence of standardized treatment guidelines presents significant diagnostic and therapeutic challenges. Currently, surgery remains the preferred treatment modality for synchronous multiple primary cancers (2). CRT is the standard nonoperative treatment for locally advanced esophageal squamous cell carcinoma (3). PD-1 inhibitors also have been widely used in advanced esophageal cancer (4–8). The addition of induction immunotherapy to CRT could improve radiosensitivity for locally advanced esophageal cancer as non-surgical treatment (9). Therefore, the complexity of surgical procedures and the high incidence of postoperative complications, especially in older patients, make comprehensive therapy centered on chemoradiotherapy a reliable non-surgical alternative (10). This case study reviews the full course of diagnosis and treatment of a patient with synchronous double primary esophageal and lung cancers. The therapeutic strategies included induction chemoimmunotherapy, CRT for esophageal cancer, and SBRT for lung cancer. These approaches underscore the safety and feasibility of systemic therapy combined with localized radiotherapy for inoperable double primary tumors, offering new perspectives for clinical practice and treatment decision-making.

## 2 Case presentation

**Figure 1:**
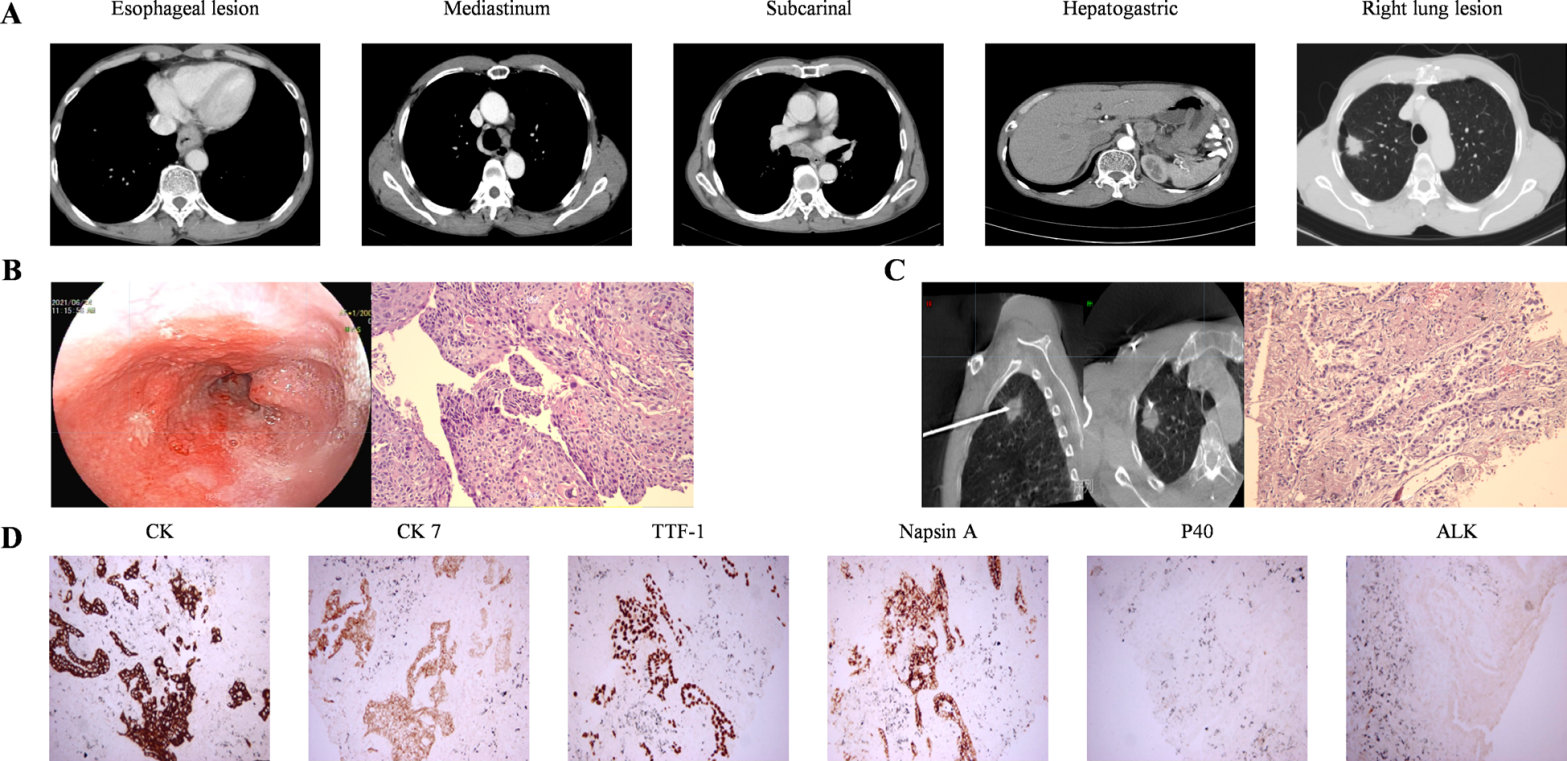
Images and pathological manifestations at baseline. **(A)** baseline CT images **(B)** Esophagoscopy findings indicated significant esophageal abnormalities. Histopathological examination of ESCC (HE ×200). **(C)** CT-guided percutaneous biopsy of the right lung lesion showed tumor cells forming acinar and tubular structures, were consistent with non-small cell carcinoma and tended to be adenocarcinoma (HE ×200). **(D)** The immunohistochemistry of lung lesion showed CK, CK7, TTF-1, and Napsin A positivity, while P40 and ALK (D5F3) were negative (200×).

A 69-year-old male patient with a 40-year smoking history (smoking index 800) presented to our hospital in June 2021. His primary complaint was progressive dysphagia for three months, which worsened to the point of tolerating only semi-liquid foods, accompanied by vomiting during meals. Esophagography with iodinated contrast revealed a 6.8 cm filling defect in the lower esophagus, along with a 2.3 cm nodule in the right upper lung field. Further evaluation with contrast-enhanced chest computerized tomography (CT) showed a 10 mm thickened wall in the lower esophagus, with multiple enlarged lymph node metastases in the mediastinum, subcarinal region, and hepato-gastric ligament, the largest measuring 26 × 24.5 mm in the hepato-gastric ligament (**Figure 1A**). A solid nodule (29 × 19 mm) was observed in the right upper lung, necessitating differentiation between primary lung cancer and metastases (**Figure 1A**). Although positron emission tomography-computed tomography (PET-CT) was recommended, the patient declined due to financial constraints. Esophageal biopsy via endoscopy confirmed moderately differentiated ESCC (**Figure 1B**). CT-guided biopsy of the right upper lung nodule confirmed the diagnosis of adenocarcinoma non-small-cell lung cancer (**Figure 1C**). The immunohistochemical analysis of lung lesion revealed positive staining for CK, CK 7, thyroid transcription factor-1 (TTF-1), Napsin A. However, P40-, ALK(D5F3) showed negative (**Figure 1D**). In lung tissue, no gene mutations were detected in lung cancer-related genetic testing, and programmed death-ligand 1 (PD-L1) was negative. After excluding mutual metastasis between the esophageal and lung tumors, the diagnosis of synchronous dual primary esophageal and lung malignancies was established. The patient had Eastern Cooperative Oncology Group Performance Status 1 (ECOG PS) and Body Surface Area 1.36 m² (BSA).

**Figure 2:**
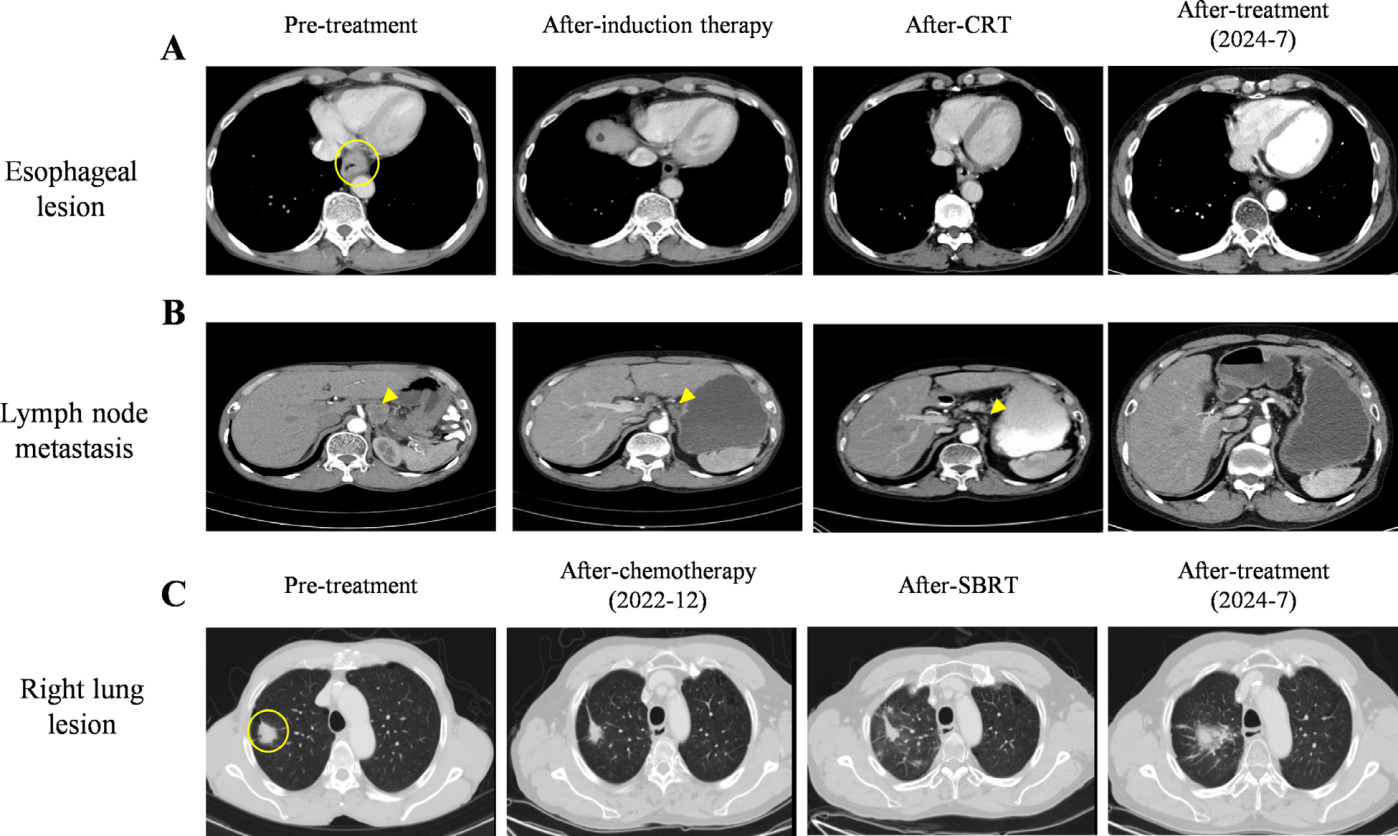
Images from pre- and after-treatment. **(A)** Pre- treatment, after-induction therapy, after-CRT, and after-treatment images of esophageal lesion on CT scans. The esophageal lesion showed a 10 mm thickened wall (○) on CT scan. **(B)** Pre- treatment, after-induction therapy, after-CRT, and after-treatment images of hepato-gastric space lymph node metastasis on CT scans. **(C)** Pre- treatment, after-chemotherapy, after-SBRT, and after-treatment images of lung lesion. Right lung lesion (29×19 mm) (○) on pre-treatment CT scan.

The patient was diagnosed with locally advanced ESCC (cT3N3M0) and adenocarcinoma non-small-cell lung cancer (cT1N0M0). When the patient was diagnosed with double primary malignant tumors, he declined surgical intervention and his nutritional status was not suitable for immediate radiotherapy. Therefore, the patient underwent two cycles of chemoimmunotherapy induction on July 9 and July 31, 2021. Each cycle included albumin-bound paclitaxel (260mg/m², intravenous drip, day 1), nedaplatin (75mg/m², intravenous drip, days 1–3), and sintilimab (anti-PD-1, 200 mg, day 1).After-induction therapy chest CT on August 25, 2021, showed improvement: esophageal wall thickening was reduced to 6 mm (previously 10 mm, **Figure 2A**), and the largest mediastinal lymph node in the hepato-gastric ligament shrank to 18 × 10 mm (previously 26 × 25 mm, **Figure 2B**). After multidisciplinary team (MDT) consultation, synchronous esophagectomy and lung resection were deemed infeasible. The patient underwent radical CRT for esophageal cancer from September 28 to November 10, 2021. Radiotherapy employed 4D-CT-guided targeting with prescription doses of gross tumor volume(GTV,56 Gy/28F), gross tumor volume of node (GTV-nd,56 Gy/28F), and clinical target volume (CTV,50.4 Gy/28F), alongside weekly albumin-bound paclitaxel (65mg/m², intravenous drip, day 1).Enhanced chest CT on November 3, 2021 (after 21 radiotherapy sessions), showed improvements: reduced esophageal narrowing, further shrinkage of hepato-gastric lymph nodes and right upper lung nodule remained unchanged at 29 × 19 mm. Subsequently, CT scans conducted in March 2022 showed an esophageal wall thickness <10mm and lymph nodes with a short axis <10mm. According to the Response Evaluation Criteria in Solid Tumors (RECIST) version 1.1, the condition was assessed as CR for the esophageal lesion. Dysphagia also significantly improved. Regular follow-ups from November 2021 to March 2022 classified the patient’s right upper lung nodule remained unchanged at 29 × 19 mm, so the pulmonary lesion as stable disease (SD).

**Figure 3:**
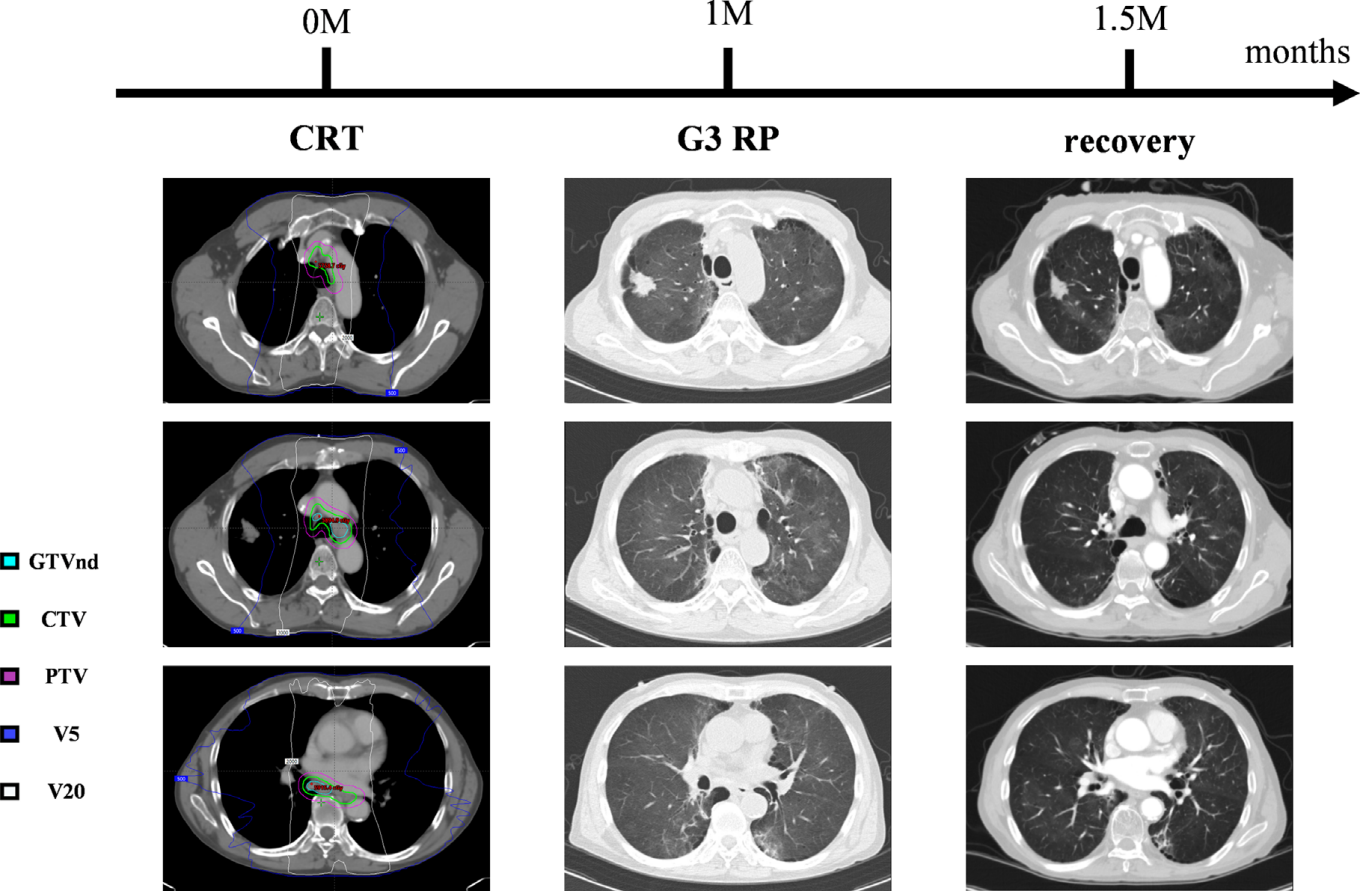
The time-series of the amelioration of radiation pneumonitis. Grade 3 RP developed one months after CRT. Dexamethasone and moxifloxacin treatment resolved the symptoms and pulmonary inflammatory shadow.

Approximately one month after completing radical chemoradiotherapy for esophageal cancer, the patient developed anorexia, fatigue, and dyspnea on exertion. Chest CT revealed new-onset pneumonia adjacent to the mediastinum in both lungs. According to the NCI Common Terminology Criteria for Adverse Events (NCI-CTCAE) version 5.0, grade 3 RP was diagnosed. The patient received dexamethasone (20mg intravenously, once daily) and moxifloxacin (0.4g intravenously, once daily) for two weeks. Follow-up CT showed significant absorption of diffuse interstitial inflammation in both lungs (**Figure 3**), with alleviation of dyspnea.

**Figure 4:**
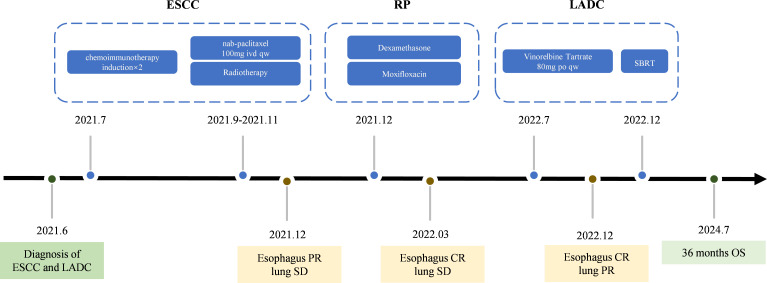
The treatment timeline.

Chemotherapy and radiotherapy were chosen because the patient refused surgery and the occurrence of RP limiting immediate SBRT treatment. In July 2022, the patient commenced antitumor chemotherapy with oral vinorelbine tartrate (80 mg, quaque-week). Follow-up imaging indicated PR of pulmonary lesions on December 2022(**Figure 2C**). Based on treatment guidelines for inoperable pulmonary nodules, stereotactic body radiation therapy (SBRT) was recommended. Following MDT discussion, radical SBRT was administered for lung cancer from December 28, 2022, to January 6, 2023, with a protocol of 50 Gy in 5 fractions (every other day). No radiation-related adverse events occurred during or after treatment. The cumulative radiation doses from both treatments are summarized in **Supplementary Table 1**. As of July 2024, chest CT evaluation showed CR for the esophageal lesion and hepatogastric lymph node metastasis (**Figures 2A, B**), PR for the lung lesion (**Figure 2C**). The patient’s entire antitumor treatment course and medication details are illustrated in **Figure 4**. By July 2024, the patient’s overall survival (OS) had reached 36 months, is currently eating well, gaining 9.0 kg of weight, and is not suffering from myelosuppression. Informed consent was obtained from the patient for the purpose of this case report.

## 3 Discussion

When two malignant tumors are diagnosed simultaneously, the primary challenge is to develop an anticancer strategy that effectively addresses both cancer types without increasing toxicity, causing pharmacological interactions, or negatively impacting overall outcomes. This case describes a rare case of synchronous double primary esophago-lung cancer. The treatment process includes induction sintilimab and chemotherapy followed by CRT for esophageal lesions and SBRT for the lung lesions. During treatment, RP was diagnosed, and through aggressive treatment and analysis, the opportunity for subsequent radiotherapy of lung lesions was obtained. The patient has survived for more than 3 years, and lung cancer was stable, meanwhile the esophageal lesion was almost cured.

Multiple primary cancer (MPC) refers to the occurrence of two or more primary malignant tumors within a single individual, either simultaneously or sequentially. These tumors may arise in different sites within the same organ or system, or in entirely distinct organs or systems. The concept was first introduced by Billroth in 1889 and later refined by Warren and Gate in 1932, who established the diagnostic criteria (11). It is necessary to use genetic examination, pathology, PEC-CT and other means before treatment identify the recurrence and metastasis of MPC (12–14).

MPC can be classified as synchronous or metachronous based on the interval between diagnoses. Tumors diagnosed within six months are defined as synchronous multiple primary cancers (SMPC), while those diagnosed after six months are classified as metachronous multiple primary cancers (MMPC) (15). These diagnostic criteria also apply to esophageal cancer with multiple primary cancers. Previous epidemiological studies report MPC incidence rates ranging from 2% to 17% (16–20). In China, hospital-based single-center studies estimate the overall incidence of MPC to be between 0.9% and 1.1% (21–23). The incidence of MPC associated with esophageal cancer is notably higher, ranging from 9.5% to 21.9% (24, 25). Moreover, Compared to the general population, individuals with esophageal cancer face a significantly increased risk of developing MPC (25). The pathogenesis of esophageal carcinoma-related multiple primary malignancies (EC-MPC) remains unclear, though certain gene mutations, such as PTEN, BRCA1/BRCA2, and CDKN2A, are associated with MPC (13). In Asian populations, primary lung adenocarcinoma patients exhibit a higher prevalence of epidermal growth factor receptor (EGFR) mutations (26). Unfortunately, in this case, EGFR and other gene mutations were not detected in the lung cancer tissue, and genetic testing was not performed on the esophageal tissue. Beyond genetic factors, endogenous influences such as sex and age also contribute to the development of esophageal MPC (27). Additionally, Hu et al. found that individuals aged 60–79 years with early-stage and/or moderately differentiated esophageal cancer were more likely to develop EC-MPC (28). Exogenous factors such as chronic exposure to tobacco and alcohol are also implicated in MPC (29–32). In this case, the patient was a 69-year-old male with a history of long-term smoking who presented with esophageal squamous cell carcinoma and lung adenocarcinoma, consistent with the clinical features of MPC. We plan to collect additional cases for further investigation into the clinical characteristics and pathogenesis of esophageal-lung multiple primary malignancies.

The treatment principle for synchronous multiple primary tumors prioritizes the more life-threatening malignancy while considering the treatment needs of all tumors. Initial treatment planning requires staging all tumors. For early-stage tumors, surgical intervention is preferred if there are no contraindications and the patient can tolerate either simultaneous or staged surgeries. For patients with contraindications or poor surgical tolerance, treatment should focus on the more aggressive tumor. To date, no standardized treatment protocol exists for MPC, though the value of surgical intervention is well-recognized. Studies indicate that for patients with esophageal cancer as the primary malignancy, concurrent surgical treatment of the second primary tumor yields better outcomes compared to other treatment modalities (33). However, surgery for MPC often demands extensive resection, poses higher procedural difficulty, and increases the risk of postoperative complications, making it highly invasive and complex, particularly when simultaneous resections are undertaken. A single-center study in China reported that 65.86% of MPC patients underwent surgery, with only 33.33% of synchronous MPC cases receiving simultaneous resections (22). Among patients achieving R0 resection, the average OS exceeded 12 months, with a recurrence rate of 24.64%. The two-year and five-year survival rates were 54.3% and 31.4%, respectively. In previously reported cases, early-stage synchronous esophageal and lung cancers were often treated with simultaneous surgical resection after thorough evaluation (34). However, in this case, the esophageal cancer was locally advanced, precluding simultaneous esophageal-lung resection.

Furthermore, surgical assessment indicated significant challenges in esophageal tumor resection. Radiotherapy, a localized cancer treatment, is generally better tolerated and more controllable than surgery. For unresectable locally advanced esophageal cancer, CRT is the standard treatment. However, the efficacy of definitive CRT remains suboptimal. With the emergence of immunotherapy, several phase I/II trials have explored combining immunotherapy with CRT for advanced, unresectable esophageal cancer. Zhang et al. evaluated a regimen combining camrelizumab with CRT in 20 patients with locally advanced ESCC, reporting 24-month OS and progression-free survival (PFS) rates of 69.6% and 65.0% (35), respectively. These outcomes surpassed the 24-month OS rate of 44.0% reported by Chen et al. for CRT without immunotherapy (36). However, the study highlighted increased adverse events associated with the addition of anti-PD-1 antibodies and higher radiotherapy doses (60 Gy and 54 Gy) (37, 38). Severe treatment-related adverse events (TRAEs) of grade ≥3 included radiation esophagitis in 20%, esophageal fistula in 10%, and TRAEs overall in 45%.Zhu et al. studied 42 untreated, unresectable stage II–IVA ESCC patients treated with toripalimab combined with definitive CRT (39). The 1-year OS and PFS rates were 78.4% and 54.5%, respectively (39). However, adverse events of grade ≥3 occurred in 86% of patients, with esophageal fistula reported in 14%. The addition of anti-PD-1 therapy to CRT may enhance survival and reduce recurrence but simultaneously increases the incidence of AEs. A single-arm, multicenter, phase 2 proof-of-concept study suggest that induction immunotherapy followed by CRT could be promote local control rate through promoting vascular normalization and alleviating hypoxia in esophageal cancer, thereby enhancing radiosensitivity (9). A propensity‐score matched study revealed the first time that induction immunotherapy plus chemotherapy followed by CRT for unresectable locally advanced ESCC provided a survival benefit with manageable safety profile (40). These findings raise important questions regarding the optimal timing of immunotherapy. Earlier intervention might prevent systemic immune effects resulting from the combination of radiotherapy and immunotherapy.

Since immune checkpoint inhibitors and radiotherapy share overlapping mechanisms of toxicity, their concurrent use may exacerbate adverse reactions. Moreover, using dual-drug chemotherapy during radiotherapy often induces significant side effects, such as bone marrow suppression. Li et al. explored the efficacy and safety of albumin -bound paclitaxel combined with radiotherapy in the treatment of ESCC, with no grade IV adverse reactions during treatment (41). In this case, our team implemented CRT (GTV 56 Gy/28F) following induction therapy with chemotherapy and immunotherapy, using paclitaxel as the sole concurrent chemotherapy agent. This approach aimed to minimize the risk of complications while rapidly controlling the local lesion. A retrospective study involving 127 patients with recurrent/metastatic ESCC who underwent immunotherapy found no statistically significant difference in OS between those who received radiotherapy and those who did not. However, among patients with locoregional recurrence, those treated with immunotherapy combined with chemotherapy followed by radiotherapy experienced a significant OS improvement (42). These findings suggest that for esophageal cancer, when systemic drug therapy proves inadequate, integrating localized radiotherapy can enhance treatment outcomes. Balancing systemic and localized therapies is crucial for effectively managing the challenges posed by tumors. However, more studies need to be done to directly address the effectiveness of non-surgical treatment strategies in esophageal double primary carcinoma.

RP is one of the most common dose-limiting toxicities associated with thoracic radiotherapy. During radiotherapy for esophageal and lung cancers, part of the lung volume is inevitably exposed to a certain dose of radiation. Increasing evidence suggests that lung injury is closely related to the low-dose volume, with higher doses to smaller lung volumes potentially being less harmful than lower doses to larger volumes. Wang et al. reported that when the low-dose volume (V5) exceeded 55% during thoracic radiotherapy, the incidence of grade ≥2 acute RP increased significantly (43). Similarly, a dosimetric analysis of CRT for esophageal cancer demonstrated a strong correlation between V5 and the occurrence of moderate-to-severe RP (44). For SBRT targeting lung lesions, planning evaluations often focus on dose constraints for V20 and mean lung dose (45). In this case, the patient developed grade 3 RP following CRT for esophageal cancer. From a dosimetric perspective, this may be attributable to an excessively high V5 dose (**Supplementary Table 1**). These findings highlight the critical importance of controlling V5 in radiotherapy planning for esophageal cancer to mitigate the risk of RP.

The management of pulmonary lesions in this case presents significant challenges. Potential treatment options include pulmonary nodule resection, SBRT, and targeted therapy. After a series of discussions, our team considered the following: the patient’s history of RP raises concerns about an increased risk of postoperative complications, such as infectious pneumonia. Genetic analysis of the patient’s lung cancer tissue showed no detectable EGFR mutations or other actionable genetic alterations, limiting the use of targeted therapies. Additionally, the patient refused surgery. For inoperable stage I–II non-small cell lung cancer, SBRT is an effective treatment modality (46). Studies and case reports on SBRT for pulmonary lesions following CRT for esophageal cancer remain scarce. A review of prospective studies on reirradiation for lung cancer highlights how advances in radiotherapy technology have made high-dose reirradiation a safer and more viable treatment option. SBRT improves accuracy in treatment planning and delivery, ensuring better target coverage while sparing organs at risk, significantly enhancing tumor control probability. After weighing the risks and benefits of various treatment options and considering the patient’s overall condition and preferences, our team implemented a stepwise treatment approach. The patient first received systemic therapy with a relatively mild oral chemotherapy regimen, followed by SBRT for the lesion in the right upper lung. This personalized treatment strategy aimed to achieve optimal therapeutic outcomes.

In conclusion, this case demonstrates a novel integrated treatment model for synchronous multiple primary lung- esophageal cancer. underscores the potential of combining systemic therapy with local radiotherapy for double primary tumors, and explores the influencing factors of thoracic radiotherapy toxicity. In the future, further prospective studies are needed to testify our findings.

## Data Availability

The original contributions presented in the study are included in the article/**Supplementary Material**, further inquiries can be directed to the corresponding author/s.

## Glossary

LADC: lung adenocarcinomas
ESCC: esophageal squamous cell carcinoma
anti-PD-1: anti-programmed death 1
CRT: definitive chemoradiotherapy
RP: radiation pneumonitis
SBRT: stereotactic body radiation therapy
PR: partial response
CT: computed tomography
PET-CT: positron emission tomography-computed tomography
TTF-1: thyroid transcription factor-1
PD-L1: programmed death-ligand 1
ECOG PS: Eastern Cooperative Oncology Group Performance Status
BSA: Body Surface Area
MDT: multidisciplinary team
GTV: gross tumor volume
GTV-nd: gross tumor volume of node
RECIST: Response Evaluation Criteria in Solid Tumors
CTV: clinical target volume
CR: complete response
SD: stable disease
NCI-CTCAE: The NCI Common Terminology Criteria for Adverse Events
MPC: multiple primary cancer
SMPC: synchronous multiple primary cancers
MMPC: metachronous multiple primary cancers
EC-MPC: esophageal carcinoma-related multiple primary malignancies
EGFR: epidermal growth factor receptor
OS: overall survival
PFS: progression-free survival
TRAEs: treatment-related adverse events
